# Outcomes of Metastatic Brain Lesions Treated with Radioactive Cs-131 Seeds after Surgery: Experience from One Institution

**DOI:** 10.7759/cureus.3075

**Published:** 2018-07-30

**Authors:** Yuanxuan Xia, Leila A Mashouf, Brock R Baker, Russell Maxwell, Chetan Bettegowda, Kristin J Redmond, Lawrence R Kleinberg, Michael Lim

**Affiliations:** 1 Neurosurgery, The Johns Hopkins University School of Medicine, Baltimore, USA; 2 Radiation Oncology, The Johns Hopkins University School of Medicine, Baltimore, USA; 3 Neurosurgery, Department of Neurosurgery/The Johns Hopkins University School of Medicine, Baltimore Maryland, Baltimore, USA; 4 Radiation Oncology and Molecular Radiation Sciences, The Johns Hopkins University School of Medicine, Baltimore, USA

**Keywords:** brachytherapy, brain metastases, local tumor control, local recurrence

## Abstract

Introduction

Brain metastases are common in patients with advanced systemic cancer and often recur despite treatment with surgical resection and radiotherapy. Whole brain radiation therapy (WBRT) and stereotactic radiosurgery (SRS) have significantly improved local control rates but are limited by complications including neurocognitive deficits and radiation necrosis. These risks can be higher in the re-irradiation setting. Brachytherapy may be an alternative method of additional targeted adjuvant radiotherapy with acceptable rates of toxicity.

Methods

A retrospective chart review of all patients undergoing resection for metastatic brain lesions and permanent low-dose rate Cs-131 brachytherapy was performed for one institution over a 10-year period. All patients had previous radiation therapy already and, after surgery, were followed with imaging every three months. Patient demographics, disease characteristics, intracranial disease, peri- and post-operative complications, and outcomes were recorded. The primary outcome of interest was local tumor recurrence at the site of brachytherapy while secondary outcomes included distant disease progression (within the brain) and complications such as radiation necrosis.

Results

During the study period, nine cases of individual patients met inclusion criteria. The median preoperative lesion diameter was 3 cm (0.8–4.1). The median overall survival after surgery and brachytherapy was 10.3 months, after excluding two patients who were lost to follow-up. Six of nine patients had no local recurrence, while three patients had development or progression of distant lesions. No patients experienced acute or delayed complications.

Conclusion

Cs-131 brachytherapy is a promising alternative method for controlling brain metastases after previous radiation interventions and surgical resection. In this case series, there were no incidences of local tumor recurrence or complications such as radiation necrosis.

## Introduction

Brain metastases are common and can occur in 25% of patients with systemic cancer [1]. Although most cancer patients succumb to complications of their systemic disease, modern therapies have improved patient survival and, as a result, have increased the risk of developing brain metastases [2]. Further, metastases to the brain have historically been considered a terminal disease stage due to their location, propensity for local recurrence, and spread throughout the central nervous system (CNS) in the setting of systemic disease.

Treatments for brain metastases often involve a combination of surgical resection and/or radiotherapy [2,3]. However, local recurrence is a continuing problem. Radiotherapy using whole brain radiation therapy (WBRT) has been employed the longest but studies have reported local recurrence rates up to 70% when WBRT has been used as monotherapy [4]. Stereotactic radiosurgery (SRS) reduced these rates of local failure to ~30% [5,6] and, when combined with WBRT, has shown rates of local control up to nearly 90–100% [5,6]. Today, focused methods such as SRS are more attractive in patients with oligometastatic brain metastases while WBRT is reserved for patients with higher burdens of intracranial disease [7]. SRS significantly limits the exposure of healthy brain tissue while delivering high doses of therapeutic radiation within a short period of time [8]. However, WBRT and SRS are both hampered by known complications in treating brain lesions. WBRT has well-described acute and long-term toxicities including blurred vision, cognitive decline, and ataxia [2,3,9,10]. SRS can be unsuitable for larger tumor volumes and recurrent or previously irradiated lesions due to an increased risk for radiation necrosis [11]. Other methods of treating lesions are warranted, especially in the recurrent setting.

Since brain metastases often recur locally and WBRT and SRS are limited in certain patient scenarios, there has been growing interest in alternative methods of focused re-irradiation. Permanent low-dose rate (LDR) brachytherapy seeds are one such option that can deliver targeted radiation to a specific site [12]. However, in contrast to SRS, LDR brachytherapy does so at a low rate over a longer period of time, and this unique approach has been shown to affect neoplastic cells while leaving healthy cells largely unharmed [12-14]. At the cellular level, the slow delivery has been shown to synchronize solid tumor neoplastic cells into radiosensitive G2 or M phases of the cell cycle, allow for tissue re-oxygenation in tumors for further radiosensitization, and leave normal cells with functional DNA repair machinery largely unharmed [9,13]. Since brain metastases have a high tendency to recur, permanent brachytherapy implants may have a role in treating these lesions. There is currently a paucity of data on the outcomes of brachytherapy for brain lesions, especially in the recurrent setting. Here, we report one institution’s experience on the outcomes of patients with brain metastases treated with Cs-131 brachytherapy after surgical resection.

## Materials and methods

Institutional review board approval from the senior author’s institution (IRB00092610) was obtained for this retrospective series. All patients with brain metastases treated by surgical resection and permanent Cs-131 LDR brachytherapy from 2007 to 2017 by the senior author were reviewed. Patient consent was not obtained due to the retrospective nature of this study. Inclusion criteria for relevant cases were patients over 18 years of age with a history of established metastatic cancer. The intent at the time of surgery was gross total resection of their brain metastases with placement of radioactive Cs-131 seeds lining the operative bed. Patient follow-up was obtained with imaging every three months after surgery. Patient demographics, disease characteristics, intracranial disease, peri- and post-operative complications, and outcomes were recorded and de-identified appropriately. The primary endpoint was local tumor recurrence, while secondary outcomes of interest included development or progression of distant (within CNS) metastatic disease and complications related to brachytherapy implantation. Local recurrence was defined as a progressively expanding lesion at the site of resection and brachytherapy seen on multiple scans. Distant progression was evaluated similarly for other sites within the CNS. Early complications include acute hemorrhage or infections; delayed complications include worsening headache, progressive neurological deficits, volume loss from atrophy or gliosis, and radiation necrosis [12]. Follow-up time was defined as the interval from the date of surgery to last clinic visit or date of death. All analyses were performed in STATA SE 14 (StataCorp, College Station, Texas) and statistical significance was defined as p ≤ 0.05.

## Results

During the study period, nine cases of individual patients met inclusion criteria. Their average age at the date of surgery was 53.8 years. Patient demographic information is summarized in Table 1. The median number of brain lesions at the time of surgery was 2 (1–7). The median preoperative lesion diameter was 3 cm (0.8–4.1). Eight of nine patients had prior treatment to the brachytherapy lesion: seven had prior resection, three had WBRT, eight had SRS, and three had both WBRT and SRS. The ninth patient had no prior treatment to the lesion treated with brachytherapy, but had prior treatment to another brain metastasis. The dosage range for previously administered WBRT dose was 25 to 35 Gy in 10 to 24 fractions and the range for SRS was 16 to 30 Gy in one to five fractions. Primary histology of these metastases included three patients with breast adenocarcinoma, two with lung (adenocarcinoma and small cell lung cancer), one with melanoma, one with uterine adenocarcinoma, one with follicular thyroid cancer, and one with colorectal adenocarcinoma.

**Table 1:** Summary of general patient demographics and clinical characteristics. WBRT: Whole brain radiation therapy; SRS: Stereotactic radiosurgery.

Characteristic	Value
Male	2
Female	7
Median Age at relevant Date of Surgery (years)	53.8 (35.3–71.1)
No. of prior intracranial lesions	
Median	2
Range	1–7
Maximum Preoperative Diameter (cm)	
Median	3.0
Range	0.8–4.1
No. with Previous Treatment to Brachytherapy Lesion	
None	1
Systemic Chemotherapy	9
Surgical Resection	7
WBRT (average dose, Gy)	3 (30.0 ± 5.0)
SRS (average dose, Gy)	8 (21.8 ± 5.4)
WBRT + SRS	3
Tumor Location	
Frontal	4
Parietal	1
Temporal	1
Occipital	3
Tumor Type	
Breast	3
Lung	2
Melanoma	1
Uterine	1
Thyroid (follicular)	1
Colorectal	1

**Figure 1:**
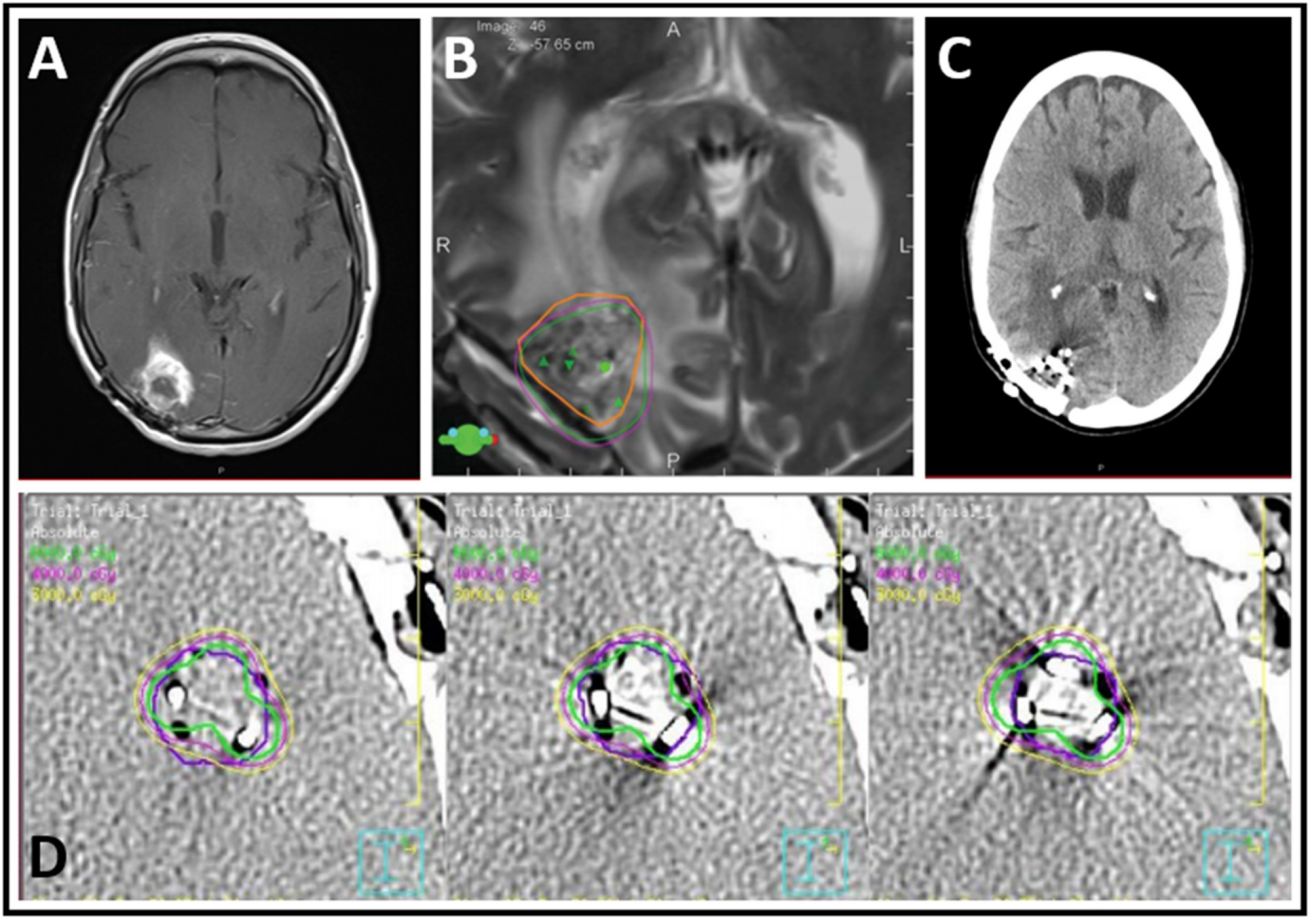
Series of images depicting preoperative, planning, and postoperative scans in a patient treated with Cs-131 brachytherapy. Preoperative T1 post-contrast magnetic resonance imaging (MRI) (A) depicts a 2.6 cm occipital lesion while post-operative T2 MRI dosimetry (B) shows the 60 Gy (purple) and 72 Gy (green) isodose lines overlayed on the planned target volume (orange). Post-operative computed tomography (CT) (C) confirmed seed placement with Leblanc dosimetry (D).

The operative parameters are reported in Table 2. The average number of Cs-131 seeds placed was 20 ± 12 with an average activity per seed of 2.6 ± 0.7 mCi at time of implantation. The average prescribed dose was 60.0 ± 3.5 Gy at depth 5 mm. Figure 1 shows the timeline of one case from preoperative imaging of the lesion to dosimetry scans and postoperative imaging.

**Table 2:** Operative parameters.

Case No.	Maximum lesion diameter (cm)	No. Cs-131 seeds placed	Activity per seed (mCi)	Prescribed dose (Gy)
1	0.8	15	2.04	55
2	4.0	30	2.04	55
3	4.1	22	1.94	60
4	2.9	4	2.14	60
5	2.7	14	2.53	60
6	2.6	18	3.15	60
7	3.3	43	2.55	60
8	3.8	23	3.54	65
9	3.0	9	3.68	65
Average		20 ± 12	2.6 ± 0.7	60.0 ± 3.5

Table 3 reports each patient’s histology and outcomes. After surgical resection and treatment with brachytherapy, none of the patients had any early or delayed complications. Six of nine patients had no recurrence, either distant or local, while three patients had distant recurrence at 1.7, 2.7, and 6.5 months from surgery. No patients had local recurrence at the treated site. By the time of data collection, two patients were lost to follow up. The median length of follow-up after surgery and brachytherapy treatment was 9.4 months (1.3–42.2). Excluding those patients lost to follow up, the median follow-up after surgery and brachytherapy was 10.3 months (6.5–42.2).

**Table 3:** Outcomes of patients treated with Cs-131 brachytherapy. ^†^Early complications include acute hemorrhage or infections; Delayed complications include worsening headache, progressive neurological deficits, volume loss from atrophy or gliosis, and radiation necrosis. ^‡^Lost to follow-up.

Case No.	Tumor histology	Complications (Early)^†^	Complications (Delayed)^†^	Tumor development^†^ (time from surgery, months)	Survival from surgery (months)
1	Lung	None	None	None	5.9^‡^
2	Breast	None	None	Distant (2.7)	14.1
3	Melanoma	None	None	Distant (1.7)	42.2
4	Breast	None	None	None	28.4
5	Colorectal	None	None	None	1.3^‡^
6	Lung	None	None	Distant (6.5)	10.3
7	Breast	None	None	None	9.4
8	Uterine	None	None	None	6.5
9	Thyroid	None	None	None	6.8

## Discussion

As the incidence of brain metastases become more frequent, new methods of delivering focused radiation are warranted to address the variety of tumors that may metastasize to the brain, especially in the setting of previous irradiation [2]. Currently, standard of care has included surgical resection and radiotherapy with either WBRT or SRS [2,3]. While SRS has supplanted WBRT for most patients with single brain metastases [4], brachytherapy is a potential alternative method for focal irradiation with promising outcomes. In this case series, no patients treated with intraoperative Cs-131 seeds developed local recurrence despite the wide variety of primary tumors, brain locations, metastatic lesion sizes, and concurrent intracranial and systemic disease burden. Further, no early or delayed complications were noted including radiation necrosis.

SRS has become the modality of choice for delivering targeted intracranial radiotherapy after surgical resection. Surgical resection alone has local control rates ranging from 45 to 60% [15,16]. However, although SRS has produced local control rates as high as 85% at one year after surgery [17], it still has shortcomings that brachytherapy may be able to address. Radiation necrosis is one common risk with rates ranging from 2-10% [18] to as high as 50% after repeat SRS to a recurrent lesion [19,20]. Radiation necrosis has been attributed to a cascade of inflammation, ischemia, and angiogenesis following endothelial damage from high-dose radiotherapies [21]. Moreover, repeat craniotomies to address them are associated with higher rates of systemic infection, worse neurological status, and depression [18].

Brachytherapy is capable of delivering a low amount of radiation over a longer period of time, potentially addressing the risk of endothelial damage and subsequent radiation necrosis caused by high dose SRS [12]. Interstitial brachytherapy seeds are often designed for therapeutic activities as low as 1 cGy/min or less over the lifetime of an implant while conventional fractionated irradiation is administered at 180–200 cGy/min spread over weeks to months [12]. Additionally, SRS is limited in larger tumors because of the higher rate of radiation necrosis, with some reporting rates up to 37.8% one year after tumors >1.5 cm are treated with SRS [22]. In this case series, tumors of different sizes were treated and almost all the lesions had been previously treated with radiation. However, no patients experienced radiation necrosis or other associated complications such as worsening headache, neurological deficits, or volume loss. Even though continued radiation after prior radiotherapy (SRS with or without WBRT) is associated with an increased risk of complications [23], especially radiation necrosis, none of the eight cases that had prior irradiation experienced post-brachytherapy complications. Multiple studies have reported similarly low rates of complications including volume loss and radiation necrosis after brachytherapy, though these studies have emphasized patients receiving only initial radiation or those treated with older I-125 radioisotopes [11,14].

Long-term local control is a key aim of adjuvant focal irradiation techniques after surgical resection. Though WBRT and SRS have significantly improved rates of local control, the results of this case series and several other reports suggest that brachytherapy is also effective in delivering robust levels of local tumor control. Wernicke et al.’s prospective trial on Cs-131 therapy after surgical resection in lesions ≥2 cm showed 100% local freedom from progression for all treated tumors regardless of size or primary cancer type [14]. Further, there was only a 7% recurrence rate within 5 mm of a resection cavity after brachytherapy placement. This trial illustrated the impressive effects of Cs-131 brachytherapy for patients but largely evaluated lesions that had not received prior irradiation. Romagna et al. compared upfront and salvage I-125 brachytherapy across 48 cases and showed local control rates of 94% and 87%, respectively [24]. Raleigh et al. showed a similar 90.5% rate of local control across 105 recurrent or large metastatic lesions for I-125 brachytherapy as well [11]. In this case series, all nine patients had complete local control and this series emphasizes the potential for Cs-131 brachytherapy in the re-irradiation setting. Cs-131 radioisotopes have been reported to have distinct radiotherapeutic advantages over I-125 isotopes including a faster half-life (9.69 days vs. 59.4 days), which may better suit an active postoperative environment and therefore be more effective [14]. Overall, the outcomes of this series are notable especially in comparison to SRS treatment, where 20–50% of brain metastases develop new or recurrent lesions within 6–12 months [25-28]. The median overall follow-up reported in this case series was 9.4 months, while the median time from the first craniotomy to diagnosed recurrence has been previously reported to be 6.7 months for patients with brain metastases [29,30].

This case series sought to report the outcomes of Cs-131 brachytherapy for a variety of metastatic brain lesions. However, this study of nine cases over 10 years is limited by the small sample size and single center experience. Additional multicenter studies incorporating larger sample sizes would be able to better define rates of local and distant control and provide a broader overview of complication rates. Further, the inherent nature of retrospective studies includes a risk of unexpected effects from unmeasured variables. Nonetheless, this case series clearly demonstrates high local control and low complications from brachytherapy in a variety of metastatic brain lesions.

## Conclusions

Brain metastases are common and account for most intracranial tumor resections. Standard of care radiotherapy often employs SRS but is limited by radiation necrosis and tumor size. Cs-131 brachytherapy is a potential alternative method for focal irradiation, especially for previously irradiated lesions. In this series, there was a remarkably high rate of local control and no reported complications including radiation necrosis.
